# Does Cluster-Robust Estimation Provide Within-Study Effects? A Comparison of Individual Participant Data Methods in MASEM

**DOI:** 10.1080/10705511.2025.2505995

**Published:** 2025-06-18

**Authors:** Lennert J. Groot, Kees Jan Kan, Suzanne Jak

**Affiliations:** University of Amsterdam

**Keywords:** IPD, meta-analysis, raw data synthesis, simulation, structural equation modeling

## Abstract

Researchers conducting meta-analytical structural equation modeling (MASEM) with individual participant data can choose from several methods, including cluster-robust estimation, two-level SEM, multivariate meta-analysis of path coefficients, and One-Stage MASEM (OSMASEM). While two-level SEM and OSMASEM model within- and between-study effects separately, cluster-robust estimation combines them, estimating an overall path coefficient. Despite its popularity, cluster-robust estimation often yields results that differ from other methods. Simulations using factor models and real-world comparisons using path models show that it may not accurately reflect within-study estimates and can produce biased standard errors. This study compares IPD MASEM methods using simulated data, varying intraclass correlations, parameter equality across levels, number of studies, and missing data. Results reveal that cluster-robust estimation frequently misrepresents within-study estimates, produces biased standard errors, and tends to incorrectly reject model fit, highlighting the need for careful method selection in IPD MASEM applications.

Adopting values put forward by the Open Science movement, increasing amounts of research data are being accrued in publicly accessible repositories (e.g., Open Science Framework). Individual participant data (IPD) meta-analysis is considered the best available procedure to synthesize these mounting sources of evidence (M. Simmonds et al., 2015; Stewart & Tierney, 2002). Most IPD meta-analyses involve univariate techniques. Univariate meta-analysis can only be used to answer a narrow selection of possible research questions that scholars may have. In order to make the most out of these available data, meta-analysts are in need of more complex tools for evidence synthesis. While approaches for multivariate meta-analysis using IPD have been proposed (e.g., Campos et al., 2023; Riley et al., 2021), validated methods for individual participant data meta-analytic structural equation modeling (IPD MASEM) are lacking. IPD MASEM is the amalgamation of IPD meta-analysis and MASEM. It distinguishes itself from traditional MASEM through the analysis of the raw data of the primary studies, rather than only the summary statistics that are usually reported in research articles.

Several analytic approaches can be taken for conducting IPD MASEM. IPD inherently has a two-level structure, with observations clustered in primary studies, so analysis options for multilevel data apply. Additionally there are options such as cluster-robust single-level SEM, multigroup SEM, multivariate meta-analysis (MVMA) of path coefficients, and correlation-based MASEM (see Groot et al., 2024). However, their solutions are not interchangeable. The decision for one method or another is to be taken, primarily, based on the specific research question. With clustered data, the research question dictates the level (or levels) at which the parameters are to be interpreted, which may vary across the available approaches (Stapleton et al., 2016). Two-level SEM explicitly separates effects at the within-study level from those at the between-study level. Multigroup SEM and correlation-based MASEM will provide estimates of (average) within- study effects. Lastly, the cluster-robust method (also referred to as ‘aggregated analysis’ or ‘design-based adjustment for clustering’) estimates overall path coefficients for a single-level model, representing a mix of within-study and between-study effects (‘total’, ‘marginal’, or ‘general’ effects).

Of the mentioned analysis approaches, cluster-robust single-level estimation is often used in practice (e.g., Blackwell et al., 2022; Huh et al., 2022; Ray et al., 2014). Cluster-robust estimation, in this context, involves adjusting standard errors and scaling the $\chi^{2}$ measure of exact fit (and $\chi^{2}$-derived measures of approximate fit). It is not always clear from the research article why researchers choose to merely adjust the standard errors and fit statistics for the clustering in their data, rather than to separate the within- and between-effects. In a meta-analytic setting, researchers often want to answer questions that involve the within-study level (i.e., the level of participants), rather than the level of the studies. Even in cases where researchers have no a priori hypotheses on study differences, it could still make sense to choose a modeling approach that can accommodate any study-mean differences that might present themselves. A lack of theory on between-study level information and lack of affinity or familiarity with multilevel modeling may be reasons why they steer towards a single-level approach such as cluster-robust estimation.

Huh et al. (2022) do describe their rationale for their decision and warrant their choice for a design-based adjustment to clustering. The paper states that multilevel regression coefficients and estimates from a single-level model both can be interpreted as marginal estimates, making design-based adjustment for clustering and model-based adjustment ‘functionally equivalent’ (p. 396). Nonetheless, warnings that the single-level parameter estimates in the context of nested data are difficult to interpret are all but new (e.g., Cronbach et al., 1976; Raudenbush & Bryk, 2002).

Wu and Kwok (2012) investigated design-based adjustment for clustering when fitting factor models, extending on the research into aggregated analysis by Muthén and Satorra (1995). Using simulations, they showed that design-based adjustment for clustering (i.e., cluster-robust estimation) only provide satisfying results when the factor structure and population values are held equal at the between and within levels. They conclude that the model-based adjustment ‘produces more consistent and efficient model parameter estimates’ (p. 33). However, their research was limited to factor models. Also, they did not investigate the effect of setting the population values of the parameters unequal across levels.

Results of evaluating path models using empirical data appear to align with the simulations of Wu and Kwok (2012). In Groot et al. (2024), a range of IPD MASEM approaches was illustrated and compared using a collection of empirical data sets (see Hagger et al., 2022). Path coefficients from methods using model-based adjustment, producing within-level estimates, were all very close to one another while the path coefficients from design-based approach, not producing within-level estimates, were notably different.

## 1. Current Study

It is evident from theory and empirical examples that cluster-robust single-level SEM parameters cannot always be expected to equal within-level estimates from other modeling approaches. Given that prior research has shown that unequal within and between-level structures can influence results when evaluating factor models (Wu & Kwok, 2012), we will investigate this premise in the context of path models, and with the inclusion of different population values across the levels. Therefore, the aim of this study is to answer the question when cluster-robust single-level estimates reflect within-study level estimates of path coefficients, and when they do not.

We aim to answer this research question by means of a Markov chain Monte Carlo (MCMC) method simulation study. We will simulate data according to several scenarios, before fitting a path model to these data collections by means of different analytical approaches. The evaluated analytical approaches, the data generating mechanism, as well as the manipulated factors, are introduced below.

### 1.1. Analysis Approaches

In this simulation study, we will evaluate the performance of different analysis approaches that can be taken to evaluate a path model in a meta-analytic context, using IPD. We focus on five different analysis approaches. These are: (a) the single-level path model with cluster-robust standard errors, (b) the partially saturated model, which is also referred to as the maximum model; (c) the two-level path model; (d) multivariate meta-analysis of path coefficients (MVMA); and (e) One-stage MASEM (OSMASEM).

#### 1.1.1. Cluster-Robust Single Level Model

The cluster-robust single-level model is the first approach of interest. It is also referred to as aggregated analysis or the single-level model with design-based adjustment for clustering (Muthén & Satorra, 1995; Wu & Kwok, 2012). The method is commonly used, as it is easy to conduct. A path model is evaluated by minimizing the misfit between the model-implied variance-covariance matrix Σ and the observed variance-covariance matrix $\hat{\Sigma }\text{.}$ The numbers of rows and columns in this matrix equal the number of variables *p* in the hypothesized model. The single-level path model has the following matrix algebraic expression:

(1) $$\Sigma ={\left(I-B\right)}^{-1}\Psi {\left(I-B\right)}^{-1T},$$

with p×p matrix B containing the path coefficients, p×p matrix Ψ containing the (residual) variances and covariances, and I representing an identity matrix. The corrections that take place in the cluster-robust procedure only affect the standard errors, by adjusting the standard errors using a sandwich estimator (Hox et al., 2018a; White, 1984), and the $\chi^{2}$ (and $\chi^{2}$-derived) fit statistics (Muthén & Satorra, 1995; Raudenbush & Bryk, 2002). The path coefficients themselves equal those obtained from the naive pooling approach, where all observations from all primary studies would be treated as independent and analyzed as one large sample. Technically, the estimates produced by this cluster-robust method are neither within-study nor between-study level estimates, but they are a function of the within and between-level parameters.

#### 1.1.2. Partially Saturated or Maximum Model

The partially saturated model (or maximum model; Hox, 2002; Hox et al., 2018b) is a two-level model that explicitly separates the within-studies (denoted by subscript W) and between-studies (denoted by subscript B) models. In a two-level model, the total covariance matrix $\Sigma_{T}$ is decomposed into a set of orthogonal covariance matrices $\Sigma_{T}=\Sigma_{W}+\Sigma_{B}$ The hypothesized model is specified on the within-study level, while the between-studies level model is saturated. In the partially saturated model, there is no path structure, and therefore no **B** matrix, at the between-study level. Hence, the matrix algebraic expression for the partially saturated path model amounts to:

(2) $$\Sigma_{T}={\left(I-B_{W}\right)}^{-1}\Psi_{W}{\left(I-B_{W}\right)}^{-1T}+\Psi_{B},$$

with the $\Psi_{B}$ matrix containing the between-study level variances and covariances equating the $\Sigma_{B}$ matrix in the absence of a B matrix at this level. In a meta-analytic setting, researchers are often interested in within-study level estimates, and this model provides estimates for the hypothesized model at the within-level, while replicating the variance-covariance matrix at the saturated between-level.

#### 1.1.3. Two-Level Path Model

The two-level path model is nested within the partially saturated model. In contrast to the partially saturated model the two-level path model also has a hypothesized structure modeled at the between-studies level. Although between-study level effects are less often part of the research questions of meta-analysts, and can be harder to interpret, they may be estimated using a full two-level SEM on the pooled IPD. The expression of a two-level path model is as follows:

(3) $$\Sigma_{T}={\left(I-B_{W}\right)}^{-1}\Psi_{W}{\left(I-B_{W}\right)}^{-1T}+{\left(I-B_{B}\right)}^{-1}\Psi_{B}{\left(I-B_{B}\right)}^{-1T},$$

where the regression coefficients in $B_{W}$ can be interpreted as a regression on the individual level, while the the regression coefficients in $B_{B}$ should be interpreted as the effect of the study mean of a variable on the study mean of another variable.

#### 1.1.4. Multivariate Meta-Analysis of Path Coefficients

Multivariate meta-analysis of path coefficients is a two-stage approach, where the path model is first evaluated in every primary study. The matrix algebraic expression of a path model for each group *j* is

(4) $$\Sigma_{j}={\left(I-B_{j}\right)}^{-1}\Psi_{j}{\left(I-B_{j}\right)}^{-1T}.$$

Next, the obtained path coefficients are meta-analyzed using multivariate meta-analysis. The meta-analytic model is expressed as:

(5) $$\begin{array}{l} {\hat{\beta }}_{j}|\beta_{j}∼N(\beta_{j},V_{j})\\ \beta_{j}∼N(\beta ,T^{2}), \end{array}$$

where ${\hat{\beta }}_{j}$ is a vector of estimated path coefficients for each of the *k* trials, normally distributed around unknown population value $\beta_{j}$ with within-study covariance matrix $V_{j}\text{.}$ The study-specific $\beta_{j}$ are in turn distributed around a vector of grand mean path coefficients β with between-study covariance matrix $T^{2}\text{.}$

#### 1.1.5. One-Stage MASEM

One-stage MASEM is a random-effects, correlation-based MASEM approach that fits the hypothesized model on averaged correlations across the primary studies. OSMASEM meta-analyzes the bivariate correlation coefficients:

(6) $$\begin{array}{l} r_{j}|\rho_{j}∼N(\rho_{j},V_{j})\\ \rho_{j}∼N(\rho_{R},T^{2}), \end{array}$$

where $r_{j}$ represents a vector of estimated correlation coefficients, normally distributed around a vector of study-specific population correlation coefficients $\rho_{j}\text{,}$ with sampling covariance matrix $V_{j}\text{.}$ Population correlation coefficients $\rho_{j}$ are normally distributed around the model-implied average correlation structure $\rho_{R}\text{,}$ with variance–covariance matrix $T^{2}$ as heterogeneity component. $\rho_{R}$ is modeled as a function of path coefficients:

(7) $$\rho_{R}=\text{vechs}({\left(I-B\right)}^{-1}\Psi {\left(I-B\right)}^{-1T}),$$

where vechs() indicates transforming the lower triangle of a matrix into a column vector (strict half-vectorization).

### 1.2. Data Generation

**Figure 1.**
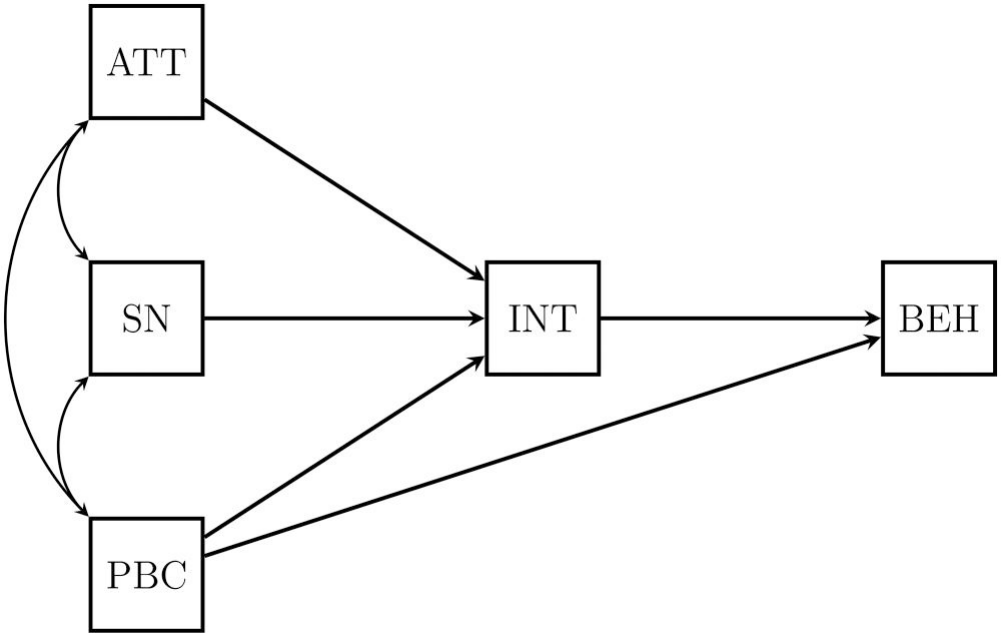
Path model for the theory of planned behavior (Ajzen, 1991). *Note.* ATT = Attitudes; SN = Subjective Norms; PBC = Perceived Behavioral Control; INT = Intentions; BEH = Behavior.

As the basis for this simulation study, we use data characteristics of Hagger et al. (2022), which contains data on health behaviors. We believe these are a good example of what typical quantitative data look like in the social and behavioral sciences, in health sciences, and economics. These data sets contain observations on five variables that can be used to evaluate a path model representing the Theory of Planned Behavior (TPB, see Figure 1; Ajzen, 1991).

The data generating mechanism followed a two-level approach, because simulating this method allows us to vary the within-study and between-study level variance-covariance structure as well as the intraclass correlation coefficients (ICCs) of the variables. Additionally, it produces a simulated data set that closely resembles a collection of IPD as would be used in practice. Such a data set contains observations of several variables for participants that are nested in primary studies.

We used a set of two variance-covariance matrices Σ—one for the between-study level ($\Sigma_{B}$) and one for the within-study level ($\Sigma_{W}$). Each of these Σ matrices, in turn are computed from their own B matrix containing regression coefficients, and a Ψ matrix containing residual (co)variances (see also Equations 1–3). First, we generated variable’s group means for each primary study from the between-level Σ. Second, the individual observations, representing the individual participant’s data in each of the primary studies, are generated for each primary study based on the group means that were generated in the first stage, and the within-level Σ matrix. Sample sizes for the primary studies are randomly sampled (with replacement) from a range of values defined by the minimum and maximum sample sizes that were reported in Hagger et al. (2022; i.e., 90≤n≤1238). This data generating mechanism allows us to manipulate a range of different factors, which we describe below.

### 1.3. Manipulated Factors

#### 1.3.1. Structural Equality and Intraclass Correlation

The matrix containing the total variance-covariance structure $\Sigma_{\text{Total}}$ is decomposed into two separate Σ matrices, one for the between-study level ($\Sigma_{B}$), and one for the within-study level ($\Sigma_{W}$). The first two factors to manipulate in the data generation are the equality of within and between-level population values leading to $\Sigma_{W}$ and $\Sigma_{B}\text{,}$ and the ICCs. We held the variance-covariance matrices equal across the two levels ($\Sigma_{B}=\Sigma_{W}\text{;}$ i.e., structural equality), or we maintained the same path model at the within and between level matrices with different parameter values across levels so that $\Sigma_{B}\neq \Sigma_{W}$ (i.e., structural inequality). We manipulated the Σ matrices indirectly by applying changes to the underlying B and Ψ matrices.

ICCs are defined as the proportion of variance that is shared among members of the same group, or in this case, participants in the same primary study. It is calculated as the proportion of the variance which exists at the between-study level, in relation to the total variance (i.e., $\frac{\sigma_{B_{1,1}}}{\sigma_{W_{1,1}}+\sigma_{B_{1,1}}}\text{.}$ In the context of two-level factor models, ICC has already been shown to influence both the fit and the stability of model parameter estimates (Wu & Kwok, 2012).

When the ICC is equal to .5, this denotes the variance is equally split among the two levels. If this is due to equal B and Ψ matrices over the levels, we call this condition ‘structural equality’. We varied the ICC by choosing different values for the B and Ψ. Considering these two factors, we determined four possible scenarios that are interesting to investigate.

##### Scenario 1: Unequal Σ Matrices and Low ICC

1.3.1.1.

For the first scenario, we use different B and Ψ across the two levels. By varying the population values we could set the ICC to take on the desired value. We chose a value of .0525 to represent low ICC. Table 1 shows the matrices containing the population values that belong to this scenario.

**Table 1.** Population values of B, Ψ, and Σ for generating the data in conditions with unequal structures and low ICC.

Within						Between				
B	ATT	SN	PBC	INT	BEH	ATT	SN	PBC	INT	BEH
ATT	0	0	0	0	0	0	0	0	0	0
SN	0	0	0	0	0	0	0	0	0	0
PBC	0	0	0	0	0	0	0	0	0	0
INT	0.43	0.16	0.31	0	0	−0.01	0.77	0.06	0	0
BEH	0	0	0.53	0.07	0	0	0	0.02	0.59	0
Ψ										
ATT	0.99	0.35	0.4	0	0	0.05	0.04	0.01	0	0
SN	0.35	0.99	0.32	0	0	0.04	0.05	0.02	0	0
PBC	0.4	0.32	0.99	0	0	0.01	0.02	0.05	0	0
INT	0	0	0	0.5	0	0	0	0	0.02	0
BEH	0	0	0	0	0.68	0	0	0	0	0.03
Σ										
ATT	0.99	0.35	0.4	0.61	0.25	0.05	0.04	0.01	0.03	0.02
SN	0.35	0.99	0.32	0.41	0.2	0.04	0.05	0.02	0.04	0.02
PBC	0.4	0.32	0.99	0.53	0.56	0.01	0.02	0.05	0.02	0.01
INT	0.61	0.41	0.53	0.99	0.35	0.03	0.04	0.02	0.05	0.03
BEH	0.25	0.2	0.56	0.35	1	0.02	0.02	0.01	0.03	0.05

##### Scenario 2: Unequal Σ Matrices and Medium ICC

1.3.1.2.

For the condition with medium sized ICC values, we used empirical values from the aforementioned Hagger et al. (2022) data (see Table 2).^1^ Note that the empirical data from which these population values are obtained were rescaled at the within-study level, which causes the variances and covariances to resemble a correlation matrix.^2^ Finally, we show the empirical ICC values, ranging from .18 to .40, in Table 3. We labeled these empirical values as medium-sized ICCs, lying in between the generated small and large ICC values from scenarios 1 and 3.

**Table 2.** Population values of B, Ψ, and Σ for generating the data in conditions with unequal structures and medium ICC.

Within						Between				
B	ATT	SN	PBC	INT	BEH	ATT	SN	PBC	INT	BEH
ATT	0	0	0	0	0	0	0	0	0	0
SN	0	0	0	0	0	0	0	0	0	0
PBC	0	0	0	0	0	0	0	0	0	0
INT	0.43	0.16	0.31	0	0	−0.01	0.64	0.07	0	0
BEH	0	0	0.53	0.07	0	0	0	0.03	0.91	0
Ψ										
ATT	0.99	0.35	0.4	0	0	0.53	0.33	0.07	0	0
SN	0.35	0.99	0.32	0	0	0.33	0.41	0.13	0	0
PBC	0.4	0.32	0.99	0	0	0.07	0.13	0.22	0	0
INT	0	0	0	0.5	0	0	0	0	0.11	0
BEH	0	0	0	0	0.68	0	0	0	0	0.44
Σ										
ATT	0.99	0.35	0.4	0.61	0.25	0.53	0.33	0.07	0.21	0.19
SN	0.35	0.99	0.32	0.41	0.2	0.33	0.41	0.13	0.27	0.25
PBC	0.4	0.32	0.99	0.53	0.56	0.07	0.13	0.22	0.1	0.1
INT	0.61	0.41	0.53	0.99	0.35	0.21	0.27	0.1	0.29	0.26
BEH	0.25	0.2	0.56	0.35	1	0.19	0.25	0.1	0.26	0.68

**Table 3.** Empirical intraclass correlation coefficients (ICC) obtained from the data published in Hagger et al. (2022).

Variable	INT	ATT	SN	PBC	BEH
ICC	0.221	0.347	0.292	0.184	0.402

*Note.* INT = Intentions, ATT = Attitudes, SN = Subjective Norms, PBC = Perceived Behavioral Control, BEH = Behavior.

##### Scenario 3: Unequal Σ Matrices and High ICC

1.3.1.3.

For high intraclass correlation, we adhere to an ICC value of 0.50. This corresponds to a scenario where the diagonal of the variance-covariance structure across the two levels is identical, indicating that exactly half of the total variance occurs at the between-study level. We created a condition where there is high ICC combined with unequal structures in the Σ matrices by specifying equal Ψ but unequal B matrices across the two levels (see Table 4).^3^

**Table 4.** Population values of B, Ψ, and Σ for generating the data in conditions with unequal structures and high ICC.

Within						Between				
B	ATT	SN	PBC	INT	BEH	ATT	SN	PBC	INT	BEH
ATT	0	0	0	0	0	0	0	0	0	0
SN	0	0	0	0	0	0	0	0	0	0
PBC	0	0	0	0	0	0	0	0	0	0
INT	0.43	0.16	0.31	0	0	−0.01	0.77	0.06	0	0
BEH	0	0	0.53	0.07	0	0	0	0.02	0.59	0
Ψ										
ATT	0.99	0.35	0.4	0	0	0.99	0.7	0.2	0	0
SN	0.35	0.99	0.32	0	0	0.7	0.99	0.43	0	0
PBC	0.4	0.32	0.99	0	0	0.2	0.43	0.99	0	0
INT	0	0	0	0.5	0	0	0	0	0.38	0
BEH	0	0	0	0	0.68	0	0	0	0	0.65
Σ										
ATT	0.99	0.35	0.4	0.61	0.25	0.99	0.7	0.2	0.54	0.32
SN	0.35	0.99	0.32	0.41	0.2	0.7	0.99	0.43	0.77	0.47
PBC	0.4	0.32	0.99	0.53	0.56	0.2	0.43	0.99	0.39	0.25
INT	0.61	0.41	0.53	0.99	0.35	0.54	0.77	0.39	0.99	0.59
BEH	0.25	0.2	0.56	0.35	1	0.32	0.47	0.25	0.59	1

##### Scenario 4: Equal Σ Matrices and High ICC

1.3.1.4

In this final scenario, the population B, Ψ, and Σ matrices were all held equal at within and between-study level. The within-study level values of the previous scenarios are now used on both levels (see Table 5). In this case, the variances are equal at the within-and between study levels. This implies, by definition, that the ICCs for the observed variables equal .50.

**Table 5.** B, Ψ, And Σ matrices for equal structures.

Within						Between				
B	ATT	SN	PBC	INT	BEH	ATT	SN	PBC	INT	BEH
ATT	0	0	0	0	0	0	0	0	0	0
SN	0	0	0	0	0	0	0	0	0	0
PBC	0	0	0	0	0	0	0	0	0	0
INT	0.43	0.16	0.31	0	0	0.43	0.16	0.31	0	0
BEH	0	0	0.53	0.07	0	0	0	0.53	0.07	0
Ψ										
ATT	0.99	0.35	0.4	0	0	0.99	0.35	0.4	0	0
SN	0.35	0.99	0.32	0	0	0.35	0.99	0.32	0	0
PBC	0.4	0.32	0.99	0	0	0.4	0.32	0.99	0	0
INT	0	0	0	0.5	0	0	0	0	0.5	0
BEH	0	0	0	0	0.68	0	0	0	0	0.68
Σ										
ATT	0.99	0.35	0.4	0.61	0.25	0.99	0.35	0.4	0.61	0.25
SN	0.35	0.99	0.32	0.41	0.2	0.35	0.99	0.32	0.41	0.2
PBC	0.4	0.32	0.99	0.53	0.56	0.4	0.32	0.99	0.53	0.56
INT	0.61	0.41	0.53	0.99	0.35	0.61	0.41	0.53	0.99	0.35
BEH	0.25	0.2	0.56	0.35	1	0.25	0.2	0.56	0.35	1

#### 1.3.2. Number of Primary Studies

Cluster-level sample size (denoted *k*) is a factor that can influence the stability of path coefficients. When conducting random-effects MASEM the number of included primary studies should ideally be at least 30 (Jak & Cheung, 2020). multilevel modeling approaches also provide more stable results when the number of clusters increases (Maas & Hox, 2005; Meuleman & Billiet, 2009) In practice, systematic reviews by M. C. Simmonds et al. (2005); M. Simmonds et al. (2015) show the number of primary studies included in published IPD meta-analyses included ranged from 2 to 78. Many IPD MA and IPD MASEM studies report study-level sample sizes of around 10 primary studies. For example, the median value found across 100 IPD meta-analyses was 8 in M. Simmonds et al. (2015). A study-level sample size of 50 could be considered large in a practical sense, but desirable in terms of power and precision. Therefore, we chose to vary the values for k=10,30,50.

#### 1.3.3. Missingness

A scenario where there is (at least some) missing data is probable in real-life applications of IPD MASEM. Missing data can occur at either the participant or the variable level. Missing data at the participant level is not particularly interesting in the context of this study, because all of the involved methods are known to be able to deal with that type of missingness. Missing data at the variable level, on the other hand, is of interest because this type of missingness is not handled equally across the methods. This condition does pose a problem for MVMA because it relies on MG SEM in the first stage, which does not accommodate missing data at the variable level.

In the context of the Theory of Planned Behavior, it is common to encounter studies that have no observations on the behavior variable. Therefore, we created conditions in which there are only four observed variables rather than five. In the conditions with missing data, we eliminated observations for the behavior variable in 50% of the primary studies. Because behavior is the outcome variable, we can still compare the direct effects of the exogenous variables on intention, even with the behavior variable missing.

In the MVMA approach, we can still obtain some comparable results. This was achieved by fitting a four variable (three paths) version of the path model in stage one (MG SEM) on the data from studies missing the behavior variable. This resulted in an incomplete data file for stage two (multivariate meta analysis), where for half of the studies there were three path coefficients present, and for half of the studies there are five. This, however, does not pose a problem, because multivariate meta-analysis can accommodate this type of incomplete data where not every study provides an equal number of effects to be synthesized.

### 1.4. Overview of the Simulation Study

In this study we simulated data according to four scenarios for structural equality and ICC, three cluster-level sample sizes *k*, and two missing data scenarios. Hence, we simulated data according to 4×3×2=24 conditions, generating 1000 collections of individual participant data in each condition. On these 24,000 meta-analytic data sets, we conducted IPD MASEM using five distinct approaches in order to evaluate the performance of each of these procedures under the different manipulated conditions. All analyses were conducted with the R (Version 4.3.3; R Core Team, 2020) programming language for statistical computing, using CRAN distributed packages MASS (Version 7.3-60.2 Venables & Ripley, 2002), lavaan (multilevel and multigroup SEM; Version 0.6-19; Rosseel, 2012), metafor (multivariate meta-anlaysis; Version 4.6-0; Viechtbauer, 2010), and metaSEM (OSMASEM; Version 1.5.0; Cheung, 2015). Annotated syntax for the data generation, model fitting, and extraction of results for this study can be found in the online supplementary materials (https://osf.io/qh4jn/). Since the within-level parameter values were not random across studies in the data-generation, we applied fixed-effects MVMA and OSMASEM.

## 2. Evaluation Criteria

After conducting IPD MASEM on all the data sets in each condition, we were primarily interested in the parameter estimates, in order to answer our main research question. Additionally we evaluated the accompanying standard errors, Root Mean Square Error (RMSE) of the estimated path coefficients, convergence rates of the analysis approaches and the obtained model fit. For evaluating the standard errors, we compute the percentage relative bias. Root Mean Square Error is a measure that can be used to compare the bias and precision of estimated path coefficients $\hat{\beta }$ across conditions and methods, given as $\text{RMSE}=\sqrt{\frac{1}{n_{\text{rep}}}\times \sum_{i=1}^{n}{\left({\hat{\beta }}_{i}-\beta \right)}^{2}}\text{,}$^4^ where values closer to zero are better. Model fit will be evaluated in terms of exact fit and approximate fit. Exact fit is rejected or not rejected based on the $\chi^{2}$-test statistic and associated *p* value. Approximate fit is assessed through the RMSEA value, by evaluating the null hypothesis for close fit $H_{0}:\text{RMSEA}<.05\text{.}$

## 3. Expectations

In terms of parameter estimates, we expected the methods that explicitly model within-study level estimates (i.e., partially saturated model, two-level model, OSMASEM and MVMA) to closely reproduce the within-study level population values. Based on earlier described studies, we expected the cluster-robust single-level estimates to differ from the within-study level population values.

Based on prior simulations, we expected that small study-level sample sizes can be problematic for convergence in multilevel modeling approaches (Hox & Maas, 2001) such as the partially saturated and two-level path model, as well as for correlation-based MASEM approaches such as OSMASEM (Jak & Cheung, 2020).

In terms of exact model fit, one would expect a rejection rate based on the $\chi^{2}$ statistic in 5% of the replications (equal to the employed criterion for α) for a correctly specified model. Given that the data-generating mechanism is a two-level model, we expected close fit to hold when fitting any of the two-level models. Even though the data-generating mechanism is two-level, we still expect similar rejection rates for exact fit with OSMASEM. This is despite the fact that this approach lacks between-study level, because it operates on the correlation matrices of each of the primary studies. Therefore, study-level averages are not part of the model and differences between study-level averages are not modeled. In a sense, that makes OSMASEM comparable to the partially saturated model, where there is no structural model at the between-study level, only a saturated covariance matrix. In terms of close or approximate fit, we would expect close fit rejected in 0% of replications based on the RMSEA, because we fit the same path model to the data that was used in the data generation.

## 4. Results

### 4.1. Convergence Rates

The convergence rates of the models under different conditions across the analysis approaches are shown in Table 6. In conditions with low convergence rates (less than 500 converged replications) results are not interpreted and compared. The convergence issues occurred in conditions with low numbers of primary studies and missing data for the multilevel modeling approaches. This partly corresponds with expectations because the number of studies under the k=10 conditions is smaller than the number of estimated parameters at the between-study level of the two-level models. There is a difference, however, among the small cluster-level sample size conditions, between those with and without missing data. In the case of missing data, lavaan, switches to a different estimation algorithm that fails to converge more often than the default algorithm that is used without missing data. In the presentation of the results for the path coefficients, standard errors, and model fit, no results are presented for the approaches under conditions with convergence rates below 50% (500 out of 1,000 replications).

**Table 6.** Convergence rates across conditions for the different IPD MASEM approaches.

Missingness	Structural equality	ICC	*k*	CR	PS	2L	OSMASEM
No missing data	$\Sigma_{B}\neq \Sigma_{W}$	Small	10	100	93.8	98.6	100
			30	100	100	100	100
			50	100	100	100	100
		Medium	10	100	100	100	100
			30	100	100	100	100
			50	100	100	100	100
		Large	10	100	99.9	100	100
			30	100	100	100	100
			50	100	100	100	100
	** ** $\Sigma_{B}=\Sigma_{W}$	Large	10	100	100	100	100
			30	100	100	100	100
			50	100	100	100	100
Missing data	** ** $\Sigma_{B}\neq \Sigma_{W}$	Small	10	100	0.0	27.0	100
			30	100	100	100	100
			50	100	100	100	100
		Medium	10	100	0.0	66.2	100
			30	100	100	100	100
			50	100	100	100	100
		Large	10	100	0.0	82.5	100
			30	100	100	100	100
			50	100	100	100	100
	** ** $\Sigma_{B}=\Sigma_{W}$	Large	10	100	0.0	85.1	100
			30	100	100	100	100
			50	100	100	100	100

*Note*. ICC = Intraclass Correlation Coefficient; *k* = cluster-level sample size; CR = Cluster-robust single-level model; PS = Partially saturated model; 2 L = Two-level model; OSMASEM = One-stage MASEM. Estimation in the (second stage of the) MVMA approach does not involve an algorithm that does or does not reach convergence and is therefore omitted from this table.

### 4.2. Path Coefficients

**Figure 2.**
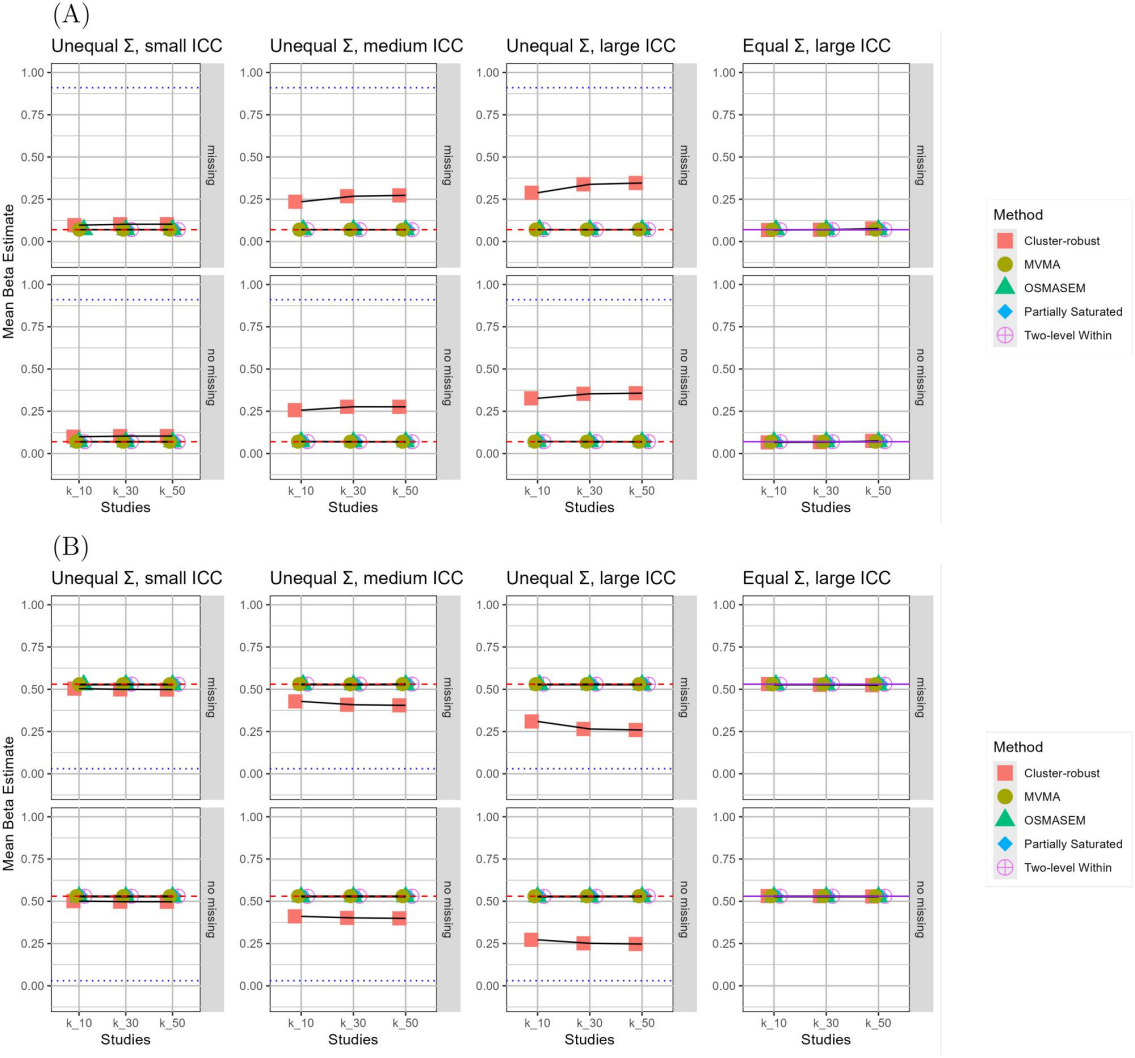
Mean estimated direct effect of (A) intentions on behavior, and (B) Perceived behavioral control on behavior across conditions per analysis approach. *Note*. Grid columns show Σ/ICC scenario’s. Rows indicate presence of missing data. Red dashed horizontal lines indicate population values for within-study level ($\beta_{\text{beh, int}}=0.07,\beta_{\text{beh, pbc}}=0.53$). Blue dotted horizontal lines indicate population values for between-study level ($\beta_{\text{beh, int}}=0.91,\beta_{\text{beh, pbc}}=0.03$). Purple solid lines represent overlapping blue and red lines. ICC = Intraclass Correlation Coefficient; MVMA = Multivariate meta-analysis; OSMASEM = One-Stage MASEM

Figure 2 shows the mean estimates of all the converged replications for direct effect of attitudes on intentions and the direct effect of intentions on behavior, respectively. Similar plots for the remaining paths are provided in the supplementary materials.

The figure shows that for the methods that estimated specifically within-study level path coefficients, the mean estimated path coefficients very closely reproduced the within-study level population values. The estimated within-study level path coefficients from the partially saturated model should be equal to those from the multilevel model, which can be seen confirmed in the figures. It should be noted that results for these approaches are missing in the k=10 conditions because of convergence problems. In several conditions, the mean estimated path coefficients for the cluster-robust single-level were found to deviate substantially from the population values of the within-study level path coefficients. The estimates from the multilevel approaches, multivariate meta-analysis and OSMASEM all appear quite robust in all of the conditions that were tested.

In the left-most columns, representing the scenario of unequal matrices and low ICC, the deviation between the cluster-robust single-level estimate and the within-study level population value was found to be negligible. However, when the ICC increases, as was the case in scenario two (medium ICC) and three (large ICC), the single-level estimates start to deviate increasingly from the within-study level population values. That is, when the between-study level population value is larger than the within-study level population value, the single-level estimate moves towards the between-study level population value, increasingly with the ICC. In the right-most column, representing scenario with equal matrices (and large ICC), the results show that the cluster-robust single-level model produces estimates similar to those from the other approaches. For the other path coefficient, similar results were observed (see).

### 4.3. Root Mean Square Error

**Figure 3.**
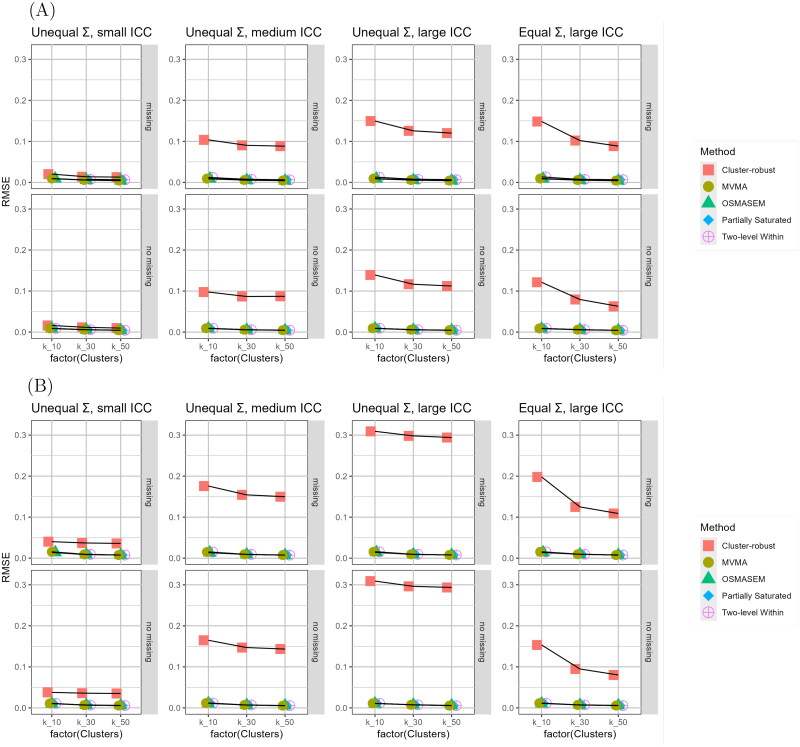
Root mean square error of parameter estimates for (A) $\beta_{\text{int,att}}$ and (B) $\beta_{\text{beh,pbc}}$ across conditions per analysis approach. *Note*. ICC = Intraclass Correlation Coefficient; MVMA = Multivariate meta-analysis; OSMASEM = One-Stage MASEM.

We calculated the Root Mean Square Error (RMSE) of each estimated path in each condition for every analysis approach. Figure 3 contains the results for one of the estimated paths. Figures containing the remaining results can be found in the supplementary materials. The RMSE values for the multilevel models, MVMA, and OSMASEM were all indistinguishably small. The RMSE values of the cluster-robust single-level model were much larger. Larger RMSE values were found in conditions with larger ICC values, either with equal or unequal Σ matrices.

### 4.4. Standard Error Bias

Table 7 shows the bias in the estimated standard errors across methods and conditions for one of the estimated paths of the evaluated model. Bias was found to be within the acceptable range of 10% throughout the chosen conditions for each of the analysis approaches, with the exception of the cluster-robust single-level model, where values were found up to five times larger than acceptable. The standard errors of the path coefficients obtained from the cluster-robust single-level model were under-estimated in most conditions, with increasing bias in conditions with missing data or smaller number of studies. The same situation was observed when looking at the results for the bias in standard errors for the additional path coefficients (see supplementary materials).

**Table 7.** Percentage bias in standard errors for $\beta_{\text{int},\text{att}}$ across conditions for the different IPD MASEM approaches.

Missingness	Structural equality	ICC	*k*	NP	CR	PS	2L-W	OSMASEM	MVMA
No missing data	$\Sigma_{B}\neq \Sigma_{W}$	Small	10	**−39.04**	**−11.87**	4.35	4.34	2.71	4.00
			30	**−42.31**	−5.63	−2.02	−2.02	−2.93	−2.51
			50	**−42.38**	−1.75	−0.42	−0.42	−2.82	−1.07
		Medium	10	**−86.52**	**−26.10**	0.38	0.38	0.61	−0.13
			30	**−86.06**	−7.83	0.90	0.90	0.88	0.46
			50	**−86.81**	−5.25	−3.76	−3.76	−4.73	−4.38
		Large	10	**−91.74**	**−28.32**	0.77	0.77	−1.71	0.30
			30	**−91.66**	**−12.85**	0.17	0.17	1.72	−0.35
			50	**−91.84**	−5.29	−3.16	−3.16	−3.16	−3.83
	$\Sigma_{B}=\Sigma_{W}$	Large	10	**−92.30**	**−26.97**	4.14	4.14	3.37	3.28
			30	**−92.74**	**−14.36**	−2.02	−2.02	−1.43	−2.61
			50	**−92.98**	−8.46	1.42	1.42	0.07	0.76
Missing data	$\Sigma_{B}\neq \Sigma_{W}$	Small	10	**−35.18**	**−15.14**			−2.87	−3.01
			30	**−41.38**	−6.34	2.55	2.55	−0.43	−1.28
			50	**−43.26**	−8.14	−1.94	−1.94	−0.24	−0.68
		Medium	10	**−85.16**	**−37.75**		5.36	1.79	1.18
			30	**−86.27**	**−14.18**	−0.29	−0.29	1.53	−0.98
			50	**−86.16**	**−10.06**	1.57	1.58	0.96	−0.87
		Large	10	**−90.60**	**−41.04**		−0.49	0.21	0.03
			30	**−91.82**	**−19.21**	−2.67	−2.67	0.05	−1.40
			50	**−91.75**	**−14.48**	−3.55	−3.55	−0.64	−1.21
	$\Sigma_{B}=\Sigma_{W}$	Large	10	**−91.36**	**−41.68**		−4.47	0.95	−1.81
			30	**−92.66**	**−19.60**	1.96	1.96	1.76	1.16
			50	**−92.83**	**−15.59**	−1.82	−1.82	3.58	2.75

*Note*. ICC = Intraclass Correlation Coefficient; *k* = cluster-level sample size; NP = Naive pooling; CR = Cluster-robust single-level model; PS = Partially saturated model; 2 L-W = Two-level model, within-study level estimates; OSMASEM = One-stage MASEM. Values represent relative bias in percentages $\frac{m_{\text{SE}}-\sigma_{b}}{\sigma_{b}}\times 100\text{.}$ Bold values exceed the threshold of acceptable bias set at 10%. Empty cells occur when convergence rate in condition was too low to compute bias.

### 4.5. Model Fit

Table 8 shows the rejection rates of the models based on the $\chi^{2}$ test of exact fit for all methods except MVMA (because this method does not provide fit measures of the meta-analytic path model). Considering the α criterion, and accounting for sampling error^5^, one would expect the percentages rejection of close fit in the range of 3.6% to 6.4% of replications. Fitting a single-level model on the combined raw data of all primary studies (naive pooling) leads to almost ineludible rejection of exact fit, due to ignoring the dependency. Results for a naive pooling single-level model are added to illustrate the correction of the $\chi^{2}$-statistic that takes place in the cluster-robust approach.

**Table 8.** Rejection rates based on $\chi^{2}$ test of exact fit across conditions and analysis approaches.

Missingness	Structural equality	ICC	*k*	NP	CR	PS	2L	OSMASEM
No missing data	$\Sigma_{B}\neq \Sigma_{W}$	Small	10	35.90	21.00	5.65	19.88	5.60
			30	44.30	16.10	4.90	7.60	5.00
			50	52.60	14.30	6.10	7.20	5.70
		Medium	10	97.20	28.20	6.20	20.00	6.00
			30	98.00	29.90	5.20	7.90	4.90
			50	97.90	29.90	5.30	6.80	5.40
		Large	10	98.10	25.00	4.80	17.70	4.80
			30	98.10	23.60	5.70	7.70	5.50
			50	98.70	19.60	4.70	6.00	4.70
	$\Sigma_{B}=\Sigma_{W}$	Large	10	98.50	25.40	5.60	16.50	6.30
			30	99.10	19.10	5.70	7.80	5.60
			50	98.90	14.70	3.80	5.70	3.70
Missing data	$\Sigma_{B}\neq \Sigma_{W}$	Small	10	32.20	20.00			5.10
			30	40.00	17.10	4.60	10.40	4.50
			50	42.10	15.40	5.00	8.00	5.20
		Medium	10	95.30	18.70		88.67	5.70
			30	97.90	27.80	4.50	13.10	4.70
			50	98.40	30.10	4.30	6.80	4.70
		Large	10	96.60	16.10		94.55	5.20
			30	97.20	23.20	5.40	10.50	5.50
			50	97.60	24.00	5.60	8.60	5.40
	$\Sigma_{B}=\Sigma_{W}$	Large	10	97.20	15.10		96.59	4.90
			30	98.70	27.80	6.50	12.70	6.50
			50	98.80	22.10	4.30	7.20	4.50

*Note*. ICC = Intraclass Correlation Coefficient; *k* = cluster-level sample size; NP = Naive pooling single-level model; CR = Cluster-robust single-level model; PS = Partially saturated model; 2 L = Two-level model; OSMASEM = One-stage MASEM. Values represent exact fit rejection rates p<α in percentages. Empty cells occur when convergence rate in condition was too low to compute rejection rates.

Regardless of the correction of the $\chi^{2}$ statistic, the rejection rates for the cluster-robust single-level path model still exceed the expected level for a correctly specified model in every condition. The results further show that the rejection rate approaches the α criterion for the partially saturated model is very close the selected level of α throughout the conditions. For the two-level model, the rejection rates approach the selected α criterion when the cluster level sample sizes are large enough. With smaller numbers of primary studies, the two-level model is rejected too often. In conditions with missing data and small number of studies the rejection rates are even larger than .80. For the partially saturated model and for OSMASEM the rejection rates fall within the confidence interval around the selected α criterion in all conditions.

Results for the evaluation of RMSEA-based close fit are reported in Table 9). For OSMASEM, the two-level model and the partially saturated model, close fit was rejected in no more than 0.1% of the replications in any condition. For the cluster-robust method, however, close fit was rejected in up to 60% of the replications. Rejection of close fit occurred mostly when cluster-level sample sizes were small, and there was missing data. Increasing ICC values corresponded with larger rejection rates.

**Table 9.** Rejection rates based on RMSEA test for close fit across conditions and analysis approaches.

Missingness	Structural equality	ICC	*k*	NP	CR	PS	2L	OSMASEM
No missing data	$\Sigma_{B}\neq \Sigma_{W}$	Small	10	0.00	10.30	0.10	0.00	0.00
			30	0.00	0.00	0.00	0.00	0.00
			50	0.00	0.00	0.00	0.00	0.00
		Medium	10	71.40	20.60	0.00	0.00	0.00
			30	62.80	1.40	0.00	0.00	0.00
			50	53.80	0.20	0.00	0.00	0.00
		Large	10	78.80	25.00	0.00	0.00	0.00
			30	65.10	1.20	0.00	0.00	0.00
			50	53.80	0.00	0.00	0.00	0.00
	$\Sigma_{B}=\Sigma_{W}$	Large	10	81.30	23.10	0.00	0.00	0.00
			30	69.90	1.00	0.00	0.00	0.00
			50	55.50	0.10	0.00	0.00	0.00
Missing data	$\Sigma_{B}\neq \Sigma_{W}$	Small	10	1.20	18.90			0.00
			30	0.00	0.60	0.00	0.00	0.00
			50	0.00	0.10	0.00	0.00	0.00
		Medium	10	72.90	49.50		0.00	0.00
			30	66.20	7.80	0.00	0.00	0.00
			50	64.70	3.80	0.00	0.00	0.00
		Large	10	82.20	56.30		0.00	0.00
			30	73.70	9.30	0.00	0.00	0.00
			50	67.90	3.50	0.00	0.00	0.00
	$\Sigma_{B}=\Sigma_{W}$	Large	10	85.00	60.90		0.00	0.00
			30	78.00	10.00	0.00	0.00	0.00
			50	75.00	3.60	0.00	0.00	0.00

*Note*. ICC = Intraclass Correlation Coefficient; *k* = cluster-level sample size; NP = Naive pooling single-level model; CR = Cluster-robust single-level model; PS = Partially saturated model; 2 L = Two-level model; OSMASEM = One-stage MASEM. Values represent percentage of replications where close fit ($H_{0}:\text{RMSEA}<0.05$) was rejected based on RMSEA *p*-values ($p_{\text{RMSEA}}\geq$ 0.05). Empty cells occur when convergence rate in condition was too low to compute rejection rates.

## 5. Discussion

We set out to answer the question when single-level path coefficients reflect within-study level path coefficients that are usually the interest of meta-analysis. The findings of this study do not support the notion that single-level estimates are similar (enough) to within-study estimates. Therefore, the design-based approach is not functionally equivalent to a model-based approach. Based on these findings we recommend using a model-based approach when answering the research question involves interpreting within-study level parameters.

In this study, we investigated the performance of different analysis approaches for evaluating a path model in meta-analytic context. We used simulations to evaluate the behaviors of five different modeling approaches in regards to estimated path coefficients, standard errors, and model fit. Generally, it can be concluded that the cluster-robust single-level can not be recommended. Single-level path coefficients did not reflect the within-study level population values in most conditions. Biases in standard errors were found to exceed thresholds of acceptability exclusively for the cluster-robust single-level model, despite the correction method that is at the core of this analysis approach. Model fit was rejected far more often than the nominal alpha level, which was not the case for other approaches. We will elaborate on these findings below, and discuss some limitations of this study, recommendations for applied researchers, and suggestions for future studies into methods for IPD MASEM.

### 5.1. Within versus Between-Level Estimates

Intuitively, one might expect that the single-level estimates would fall somewhere in between the within-study and between-study level estimates. In our study, thus was indeed the pattern that we observed. However, this is not always the case because the single-level bivariate correlation coefficient is a weighted sum (weighted by the ICC and cluster-size), rather than an average of the within-study level and between-study level coefficients (Hamaker, 2024). Path coefficients are often a complex function of several bivariate correlation coefficients, so they may behave even more erratic than the bivariate correlations.

### 5.2. When Are Cluster-Robust Single Level Models in IPD MASEM Safe to Use?

Under a very narrow set of circumstances the use of a single-level cluster robust model could be a tenable approach to synthesize IPD. These circumstances would be that there is a large number of primary studies, with little, or no missing data. Furthermore, the variables of concern all demonstrate very small ICC values, the researcher has no interest in parameter estimate’s significance (SEs), the researcher has no interest in model fit. The latter could be the case when the researchers are fitting a saturated model. Still, the disadvantages and limited practicality of the cluster-robust single-level approach, make it hard to recommend this analysis approach over its alternatives using model-based adjustments for clustering.

### 5.3. Study-Mean Centering

A possible alternative solution to using any of the discussed model-based methods is using study-mean centered data. Study mean-centered data will give single-level estimates equal to within-level estimates of two-level model, solving the main issue that comes with single-level cluster robust estimation. We did not evaluate this as a main approach because we could not find examples of this method being used in an IPD MASEM context. However, the approach has been discussed in methodological literature (Asparouhov & Muthén, 2019; Hamaker & Muthén, 2020; Lüdtke et al., 2008). Therefore, we conducted additional analyses evaluating cluster-robust single-level modeling with study-centered data. The detailed results can be found in the supplementary materials. We found that, even though the method indeed leads to within-level estimates of the path coefficients, the bias in standard errors exceeded the acceptable threshold when the number of studies was small and the Σ matrices were equal across levels. Regarding model fit, the rejection of exact fit based on the $\chi^{2}$ still occurs more often than expected based on the α criterion, and more often than for the partially saturated model and OSMASEM.

### 5.4. Limitations and Future Directions

There are several limitations to this study that could be addressed. First, there were a limited number of conditions. While we believe these to cover the most interesting and common scenarios that might present themselves in IPD MASEM context, perhaps some questions remain that cannot be answered with these simulations.

For example, we only investigated the evaluation of a path model. Although factor models have already been studied with similar findings, we did not study full SEM (models with both latent and observed variables) or any other special applications of SEM. However, we have no reason to assume the single-level cluster robust model would perform better than it did for path models or factor models in any of these situations.

Overall, the different model-based approaches that can be taken when conducting IPD MASEM, performed well. There seems to be one clear limiting factor to using most of these methods, which is the number of studies. Future research could focus on adapting or developing model-based IPD MASEM approaches that can be used when the number of primary studies with raw data is low.

An ongoing problem in the field of evidence synthesis is that researchers are unclear about their choice of analysis approach or select sub-optimal procedures (M. Simmonds et al., 2015; M. C. Simmonds et al., 2005). Examples of this are defaulting to one-stage as opposed to two stage meta-analysis (as noted by Riley et al., 2023), and the use of fixed-effects instead of random-effects modeling approaches (as noted by Wilcox & Wang, 2023). This is a phenomenon with perhaps no better explanation than precedent set by prior publications or lack of knowledge of other, more preferable, methods (Riley et al., 2010; M. Simmonds et al., 2015; M. C. Simmonds et al., 2005). The same can be stated for for MASEM applications (e.g., persistent use of univariate methods as opposed to multivariate methods; Jak & Cheung, 2024). Therefore, it is important to develop a rigorous methodological foundation for the further development of IPD MASEM, now that it is still relatively in its infancy.

Warnings against interpreting single-level regression coefficients in the context of nested data are not new (e.g., Cronbach et al., 1976; Raudenbush & Bryk, 2002). Also in the context of evidence synthesis there are many warnings against using single-level models to evaluate IPD see (Hua et al., 2017; Wilcox & Wang, 2023). Based on these simulations, we wish to extend this warning to IPD MASEM. Not only are there problems with the interpretation of the parameter estimates when using a cluster-robust single-level model, but the rejection rates and standard errors also fall outside of any acceptable thresholds.

## 6. Conclusion

In addition to the discouraging findings for use of the cluster-robust single-level approach for conducting IPD MASEM, this study has provided support for the robustness of the other evaluated approaches. The multilevel modeling approaches, as well as the multivariate meta-analysis, and correlation based OSMASEM has been shown to be quite robust in the investigated conditions. The number of studies turned out to be an important factor, given the convergence problems and inflated test statistics that can occur with multilevel modeling approaches in small sample conditions.

## Data Availability

Data, syntax, and output for analyses conducted in this research are available on the Open Science Framework (https://osf.io/qh4jn/).

## Footnotes

1 We obtained the population values of the model parameters for the data generating mechanism by fitting a two-level model on (standardized) empirical data published in Hagger et al. (2022), using lavaan (Rosseel, 2012). After fitting the two-level path model, we extracted B and Ψ matrices for both the within and between level, from which we computed the model implied Σ matrices.

2 One would expect the values along the diagonal to equal 1 as they are standardized variances, but the values obtained through the estimation procedure sometimes showed a negligible deviance from 1.

3 Because the ICC only depends on the values on the diagonal of the Σ matrices, the off-diagonal values of Σ are allowed to vary through a different structure in the B matrix.

4 Or, equivalently: $\sqrt{{\left(\bar{\beta }-\beta \right)}^{2}+\text{VAR}(\hat{\beta })}$

5 $p\pm 1.96\sqrt{\frac{\left(p(1-p\right)}{n}}\text{,}$ where *p* is the selected α criterion of .05 and the number of replications n=1000
